# Efficacy and safety of polyethylene glycol loxenatide in type 2 diabetic patients: a systematic review and meta-analysis of randomized controlled trials

**DOI:** 10.1038/s41598-023-46274-x

**Published:** 2023-11-03

**Authors:** Hazem Mohamed Salamah, Ahmed Marey, Esraa Elsayed, Mohammed Tarek Hasan, Abdelrahman Mahmoud, Khaled Alsayed Abualkhair, Dina Essam Abo-elnour, Ibrahim Abdelmonaem Abdelhaleem, Mohamed Abd-Elgawad

**Affiliations:** 1 Faculty of Medicine, Zagazig University, Zagazig, 44519 Egypt; 2 Faculty of Medicine, Alexandria University, Alexandria, Egypt; 3 Faculty of Medicine Al, Azhar University, Cairo, Egypt; 4 Faculty of Medicine, Minia University, Minya, Egypt; 5 Faculty of Medicine, Fayoum University, Fayoum, Egypt

**Keywords:** Type 2 diabetes, Drug development, Outcomes research, Clinical trial design

## Abstract

Polyethylene glycol loxenatide (PEX168) is a novel glucagon-like peptide-1 receptor agonist with a longer half-life developed by modifying the chemical structure of exenatide. This study aims to assess the efficacy and safety of PEX168 and determine the best dose. We searched PubMed, Scopus, Cochrane Library, and Web of Science databases from inception to April 25, 2023, for randomized controlled trials (RCTs) comparing PEX168 therapy alone or in combination with metformin versus other therapies. We used the risk ratio (RR) for dichotomous outcomes and the mean difference (MD) for continuous outcomes, both with 95% confidence intervals (CI). Six RCTs, including 1248 participants, were included. PEX168 added to metformin was significantly better than metformin alone regarding fasting blood glucose (MD = −1.20, 95% CI (−1.78,  − 0.62), *p* < 0.0001), HbA1c (MD = −1.01, 95% CI (−1.48,  − 0.53), *p* < 0.0001), and postprandial glycemia (MD = −1.94, 95% CI (−2.99,  − 0.90), p = 0.0003). Similarly, for glycemic control, PEX168 monotherapy was superior to placebo (*P* < 0.05). No significant effects were noticed in terms of triglycerides, low-density lipoprotein, or high-density lipoprotein (*p* > 0.05). Body weight was significantly reduced in obese diabetic patients receiving PEX168 compared to the control group (MD = −5.46, 95% CI (−7.90,  − 3.01), *p* < 0.0001) but not in non-obese patients (MD = 0.06, 95% CI (−0.47, 0.59), *p* = 0.83). People who received PEX168 alone or with metformin showed more common gastrointestinal adverse effects, especially nausea and vomiting (*p* < 0.05). PEX168 100, 200, and 300 ug monotherapy demonstrated comparable safety and diabetes control to metformin, but when combined with metformin, PEX168 100 and 200 ug showed significant effects on diabetes control; however, only the latter showed a significantly higher incidence of nausea and vomiting (*p* < 0.05). PEX168 could be a viable option for treating diabetic patients whose metformin control is inadequate or who cannot tolerate metformin. PEX168 at 100 ug in combination with metformin was found to be safe and more effective compared to metformin; however, due to the small number of trials included, these findings should be interpreted with caution, and additional trials are required.

## Introduction

Diabetes mellitus is the most common endocrine disorder in the world. In 2021, the International Diabetes Federation (IDF) estimated that the global diabetes prevalence was 10.5% (536.6 million people)^1^, which is expected to rise further in the coming years^1^. Obesity is another common condition that affects more than one billion of the world's population, according to the WHO^2^. Diabetes and obesity can have a wide range of debilitating consequences, including but not limited to cardiovascular diseases like hypertension, cardiac insufficiency, and coronary heart disease, as well as neurological diseases, renal failure, recurrent infections, retinopathy, and digestive disorders^3, 4^. Obesity-related deaths were estimated to be five million worldwide in 2019^5^, with diabetes contributing to 6.7 million deaths globally in 2021^6^.

Furthermore, diabetes and obesity-related healthcare costs are becoming an essential public and clinical health challenge. Diabetes-related global health spending was estimated to be 966 billion USD, while the economic impact of obesity was estimated at 2.19% of global gross domestic product^1, 7^. Obese patients' annual healthcare costs were found to be 100% higher than their normal-weight peers^8^, while diabetic patients spent 2.3 times more on medical care than non-diabetic patients^9^.

There are many treatment options for each of the two conditions. Drugs such as orlistat, phentermine-topiramate, and naltrexone-bupropion, as well as bariatric surgery, can be used for the management of obesity. Insulin, metformin, and sulfonylurea are available options for managing DM^10, 11^. In both conditions, lifestyle modification is essential for achieving effective management. Diet and exercise can result in a 0.3 to 2.0% decrease in HbA1c and medication doses, a 7% reduction in body weight, and a lower risk of adverse events^12–14^.

Obese men and women have a sevenfold and 12-fold increase in the risk of developing diabetes, respectively^15^. This association could be explained by the fact that obesity causes an increase in the release of fatty acids from adipose tissues, which has been linked to the development of insulin resistance and beta-cell dysfunction^16^. Therefore, there is a strong link between obesity and type 2 diabetes mellitus (T2DM), with approximately 90% of type 2 diabetic patients being obese or overweight^17^. Together, they increase the individuals' mortality risk sevenfold^18^. Common diabetes medications, such as insulin and sulfonylurea, can lead to weight gain^17^.

Glucagon-like peptide 1 receptor agonists (GLP-1RAs) are a unique class of drugs that can treat diabetes and obesity. They activate GLP-1 receptors, which are found throughout the body and serve various biological functions. GLP-1RAs work in the pancreas by stimulating GLP-1 receptors to increase insulin release, thereby alleviating hyperglycemia; in the hypothalamus, stimulation of GLP-1 receptors decreases appetite and increases satiety^19, 20^. GLP-1RAs that are commonly used include liraglutide and exenatide. They outperformed other classes of anti-diabetic drugs in terms of glycemic control and reduced cardiovascular outcomes and all-cause mortality in diabetic patients^21^. However, liraglutide and exenatide are short-acting GLP-1RAs that must be administered once daily and twice daily, respectively, which is inconvenient for long-term compliance.

Furthermore, a study found that long-acting exenatide improved glycemic control significantly more than short-acting exenatide, with similar effects on body weight and the incidence of hypoglycemia^22^. Once-weekly taspoglutide, a long-acting GLP-1RA, provided better glycemic control than short-acting exenatide but caused unacceptable adverse effects^23^. Therefore, new long-acting GLP-1 receptor agonists are required.

Polyethylene glycol loxenatide (PEX168) is a novel member of the GLP-1RAs class derived from exenatide; however, the presence of polyethylene glycol (PEG) in its structure slowed peptide chain degradation, reducing toxicity, extending the duration of action and half-life, and making it less immunogenic^24^. PEX168 is administered once a week, and patients tolerate it well^23^. Furthermore, Chen et al.^25^ found that PEX improved all glycemic control parameters, including HbA1c and fasting and postprandial plasma glucose, while maintaining excellent safety and tolerance in diabetic patients. Several trials have been conducted to assess the efficacy and safety of various doses of PEX168 in type 2 diabetic patients, either alone or as an add-on therapy^24–29^. Therefore, we performed a meta-analysis to summarize the efficacy and safety of PEX168 monotherapy or as an add-on therapy to metformin in type 2 diabetic patients. We used network meta-analysis as well to find the optimal dose.

## Methods

Our review protocol was submitted prospectively and published in PROSPERO under the ID CRD42023403563. We carried out a systematic review and meta-analysis in accordance with the Preferred Reporting Items for Systematic Reviews and Meta-Analyses (PRISMA) statement^30^.

### Literature search strategy

Web of Science, SCOPUS, PubMed, and Cochrane Central were thoroughly searched using the following search strategy: "PEX168" OR "PEX 168" OR "PEX-168" OR "PEG-Loxe" OR "PEG-loxenatide" OR "polyethylene glycol loxenatide" OR "loxenatide" in all fields through February 23, 2023. No search filters were used. Table S1 shows the entire search strategy and the results for each database. The references of the eligible papers were also searched for other relevant studies.

### Study selection and eligibility criteria

Two independent authors manually screened the retrieved records in two steps: title and abstract screening, followed by full-text screening. A third author was consulted for any discrepancies. We chose prospective randomized controlled trials (RCTs) that compared polyethylene glycol loxenatide as monotherapy or add-on therapy versus placebo, metformin, or any other comparator for patients with diabetes mellitus type 2 with or without obesity or overweight. Animal studies, in vitro studies, non-English studies, and conference abstracts were excluded.

### Data extraction

The lead author prepared formatted Excel sheets including demographic data and study characteristics, risk of bias (ROB) assessment, and outcomes of interest. Two reviewers independently extracted the following data from the eligible articles: study characteristics, including study ID, country (center), trial registry, study design, number and diagnosis of participants, intervention, and control regimens; baseline information, including age, gender, weight, BMI, fasting blood glucose (FBG), HBA1C, and diabetes duration; and the outcomes of interest. Our outcomes of interest are FBG, HBA1C, 2-h postprandial plasma glucose (PPG), body weight, triglyceride (TG), high-density lipoprotein (HDL), low-density lipoprotein (LDL), and safety outcomes such as any adverse events (AEs), nausea, vomiting, diarrhea, and discontinuation due to AEs. Disagreements were resolved through consensus.

### Risk of bias assessment

The Cochrane risk-of-bias tool, version 2^31^ was used to assess the quality of the included studies in the following domains: (A) bias arising from the randomization process; (B) bias due to deviations from intended interventions; (C) bias due to missing outcome data; (D) bias in the measurement of outcomes; and (E) bias in the selection of the reported results. The domains were assessed as either low risk, some concern, or high risk. The assessment was conducted independently by two authors, with a discussion with a third author in case of conflicts.

### Statistical analysis

We conducted a pairwise meta-analysis using RevMan version 5.4^32^. Studies were grouped according to whether PEX168 was given alone or in combination with metformin and whether the control group received a placebo or metformin. We combined all PEX168 doses in the pairwise meta-analysis. All data were collected as means ± standard deviation (SD) or event and total for continuous and dichotomous outcomes. We calculated the pooled risk ratio (RR) and 95% confidence interval (CI) for dichotomous variables and mean differences with a 95% CI for continuous variables. The statistical heterogeneity was assessed using I^2^ and the chi-squared test. The heterogeneity was considered significant if *p* < 0.1 or I^2^ > 60%. We used a random effect model to account for the heterogeneity. We conducted a subgroup analysis to determine the effect of PEX168 on weight loss in obese versus non-obese subjects.

In the network meta-analysis, a frequentist network meta-analysis was used to evaluate the efficacy and safety and rank the various PEX168 doses, and the "netsplit" function was used to split direct and indirect evidence in the network meta-analysis. The pooled mean difference and risk ratio with their 95% confidence intervals were calculated using R Studio version 1.4.1717 and the "netmeta" package. Heterogeneity was assessed using the I^2^ statistics, and a random-effects model was used when heterogeneity was present.

A funnel plot and Egger's and Begg's tests were performed to examine publication bias, and *P* < 0.05 was considered significant^33, 34^.

## Results

### Literature search results

**Figure 1:**
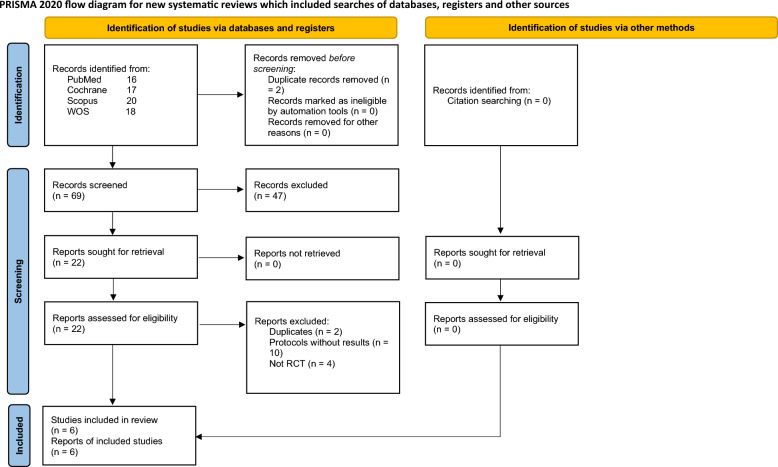
PRISMA flow diagram shows the detailed process of search strategy results and study selection. From: Page MJ, McKenzie JE, Bossuyt PM, Boutron I, Hoffmann TC, Mulrow CD et al. The PRISMA 2020 statement: an updated guideline for reporting systematic reviews. BMJ 2021:372:n71. doi: 10.1136/bmj.n71. For more information, visit: http://www.prosma-statement.org/.

A total of 71 records were initially retrieved. Two duplicates were excluded, and the remaining records were manually screened for eligibility criteria. Finally, six RCTs met the eligibility criteria and were included in our study^24–29^. Figure 1 represents the PRISMA flow diagram, which shows the detailed search strategy results and study selection process.

### Characteristics of the included studies

The total number of patients who received PEX168 was 826, distributed as follows: 374 patients were given PEX168 at 100 ug, 340 were given PEX168 at 200 ug, and 112 were given PEX168 at 300 ug, while 422 were in the control group. Four trials were multicenter^24, 25, 27, 29^, whereas two were single-center trials^26, 28^. In four trials^24, 25, 27, 29^, the participants were diabetic, and in two trials^26, 28^, participants were only included if they were diabetic with obesity. PEX168 was administered as monotherapy in three trials^24, 26, 29^ and in combination with metformin in three trials^25, 27, 28^. The control group received metformin in four trials^25–28^ and only placebo in two trials^24, 29^. The detailed study characteristics and participants' baseline data are presented in Tables 1 and 2, respectively.

**Table 1:** Summary of the included studies.

Study ID	Trial registry	Country	Study Design (arms)	Total (diagnosis)	Follow up	Intervention group	Control group	Outcomes
Chen et al.^25^	NCT01965509	China (Multicenter)	RCT (3)	118 (T2DM)	12 weeks	PEX168 100 ug and 200 ug, SC, once a week (combined with metformin 1500 mg daily)	Metformin 1500 mg daily	HbA1c, FPG, PPG, TG, HDL, LDL, safety
Gao et al.^27^	NCT02477969	China (Multicenter)	RCT (3)	533 (T2DM)	24 weeks	PEX168 100 ug and 200 ug, SC, once a week (combined with metformin 1500 mg daily)	Metformin 1500 mg daily	HbA1c, FPG, PPG, body weight, TG, HDL, LDL, safety
Shuai et al.^29^	NCT02477865	China (Multicenter)	RCT (3)	361 (T2DM)	24 weeks	PEX168 100 ug and 200 ug, SC, once a week (monotherapy)	Placebo	HbA1c, FPG, PPG, body weight, TG, HDL, LDL, safety
Li et al.^28^	ChiCTR1900026514	China (single-center)	RCT (2)	40 (T2DM and obesity)	12 weeks	PEX168 100 ug, SC, once a week (combined with metformin 1500 mg daily)	Metformin 1500 mg daily	FPG, PPG, body weight, TG, HDL, LDL
Cai et al.^26^	ChiCTR2200057800	China (single-center)	RCT (2)	156 (T2DM and obesity)	16 weeks	PEX168 300 ug, SC, once a week (Monotherapy)	Metformin 1500 mg daily	HbA1c, FPG, body weight, TG, HDL, LDL, safety
Yang et al.^24^	NA	China (Multicenter)	RCT (4)	50 (T2DM)	8 weeks	PEX168 100 ug, 200 ug, and 300 ug, SC, once a week (monotherapy)	Placebo	HbA1c, FPG, PPG, safety

*FPG* Fasting plasma glucose; *HbA1c* Glycated hemoglobin; *HDL* High-density lipoprotein; *LDL* Low-density lipoprotein; *NA* Not applicable, *PPG* Postprandial glucose; *RCT* Randomized clinical trial; *SC* Subcutaneous; *TG* Triglyceride; *T2DM* Type 2 diabetes mellitus.

**Table 2:** Baseline characteristics of the included studies.

Study ID	Groups	Sample size	Age	Female	Diabetes duration (y)	Weight (kg)	BMI (kg/m^2^)	HbA1c (%)	FPG (mmol/L)	TG (mmol/L)
			Mean ± SD	N (%)	Mean ± SD	Mean ± SD	Mean ± SD	Mean ± SD	Mean ± SD	Mean ± SD
Chen et al.^25^	PEX168 100 ug	41	52.6 ± 8.4	19 (46.34)	4.42 ± 6.42	NA	27.2 ± 3.6	8.23 ± 0.88	9.54 ± 2.42	NA
	PEX168 200 ug	39	49.8 ± 10.9	17 (43.59)	4 ± 5.67	NA	26.3 ± 3.3	8.34 ± 1.15	9.29 ± 2.33	NA
	Control	38	53.5 ± 10.2	12 (31.58)	6.46 ± 7.92	NA	27.2 ± 4.5	8.28 ± 1.05	9.47 ± 2.52	NA
Gao et al.^27^	PEX168 100 ug	179	53.6 ± 10.5	77 (43.0)	4.3 ± 3.5	71.2 ± 12.8	26.0 ± 3.5	8.5 ± 0.9	NA	1.9 ± 1.1
	PEX168 200 ug	175	52.8 ± 10.6	69 (39.4)	4.8 ± 3.2	73.6 ± 14.6	26.6 ± 3.8	8.5 ± 0.9	NA	1.9 ± 1.2
	Control	179	52.3 ± 10.7	81 (45.3)	4.7 ± 3.4	73.8 ± 14.1	26.9 ± 3.9	8.6 ± 0.9	NA	1.9 ± 1.3
Shuai et al.^29^	PEX168 100 ug	124	50.5 ± 10.4	41 (33.1)	1.85 ± 2.8	74.3 (12.7)	27.0 (3.7)	8.5 ± 0.9	9.06 ± 2.11	NA
	PEX168 200 ug	116	52.4 ± 11.5	52 (44.8)	2.27 ± 3.37	71.9 (11.2)	26.4 (3.3)	8.5 ± 0.9	9.17 ± 2.16	NA
	Control	121	51.5 ± 10.9	33 (27.3)	2.57 ± 3.9	72.8 (12.7)	26.3 (3.4)	8.6 ± 1.0	9.91 ± 2.54	NA
Li et al.^28^	PEX168 100 ug	20	63.29 ± 1.27	10 (50)	8.9 ± 1.7	NA	NA	NA	6.2 ± 1.8	2.75 ± 0.62
	Control	20	64.23 ± 1.31	10 (50)	9.9 ± 1.9	NA	NA	NA	6.4 ± 1.5	2.67 ± 0.59
Cai et al.^26^	PEX168 300 ug	104	40.3 ± 10.0	37 (35.6)	2.1 ± 1.7	87.6 (13.9)	30.0 (3.6)	8.79 ± 0.83	8.56 ± 0.87	1.88 ± 0.53
	Control	52	42.2 ± 9.5	20 (38.5)	2.1 ± 1.5	87.6 (13.9)	30.1 (3.5)	8.68 ± 0.95	8.46 ± 0.96	1.79 ± 0.52
Yang et al.^24^	PEX168 100 ug	10	53.3 ± 6.2	7 (14)	NA	63.0 ± 11.8	24.1 ± 2.2	8.0 ± 1.1	8.4 ± 1.7	NA
	PEX168 200 ug	10	48.3 ± 10.4	3 (6)	NA	73.3 ± 18.3	25.7 ± 3.7	8.7 ± 1.1	9.3 ± 2.0	NA
	PEX168 300 ug	8	52.0 ± 5.9	4 (8)	NA	66.3 ± 10.1	25.1 ± 3.7	8.0 ± 1.4	8.6 ± 2.1	NA
	Control	12	50.3 ± 10.8	2 (4)	NA	67.6 ± 12.8	24.8 ± 3.9	8.2 ± 1.2	8.2 ± 1.4	NA

*BMI* Body mass index; *HbA1c* Glycated hemoglobin; *FPG* Fasting plasma glucose; *NA* Not applicable; *TG* Triglyceride.

### Risk of bias assessment

The quality of the included studies was assessed using the revised Cochrane risk of bias tool, as shown in supplementary Fig. 1. Three trials^25, 27, 29^ had a low risk of bias in all domains. There was no information regarding allocation concealment in two trials^24, 28^. Yang et al.^24^ had a high risk of bias resulting from missing outcome data and deviation from the intended intervention. There were some concerns regarding the selection of the reported results in three trials^24, 26, 28^.

### Pairwise meta-analysis findings

#### Glycemic control

**Figure 2:**
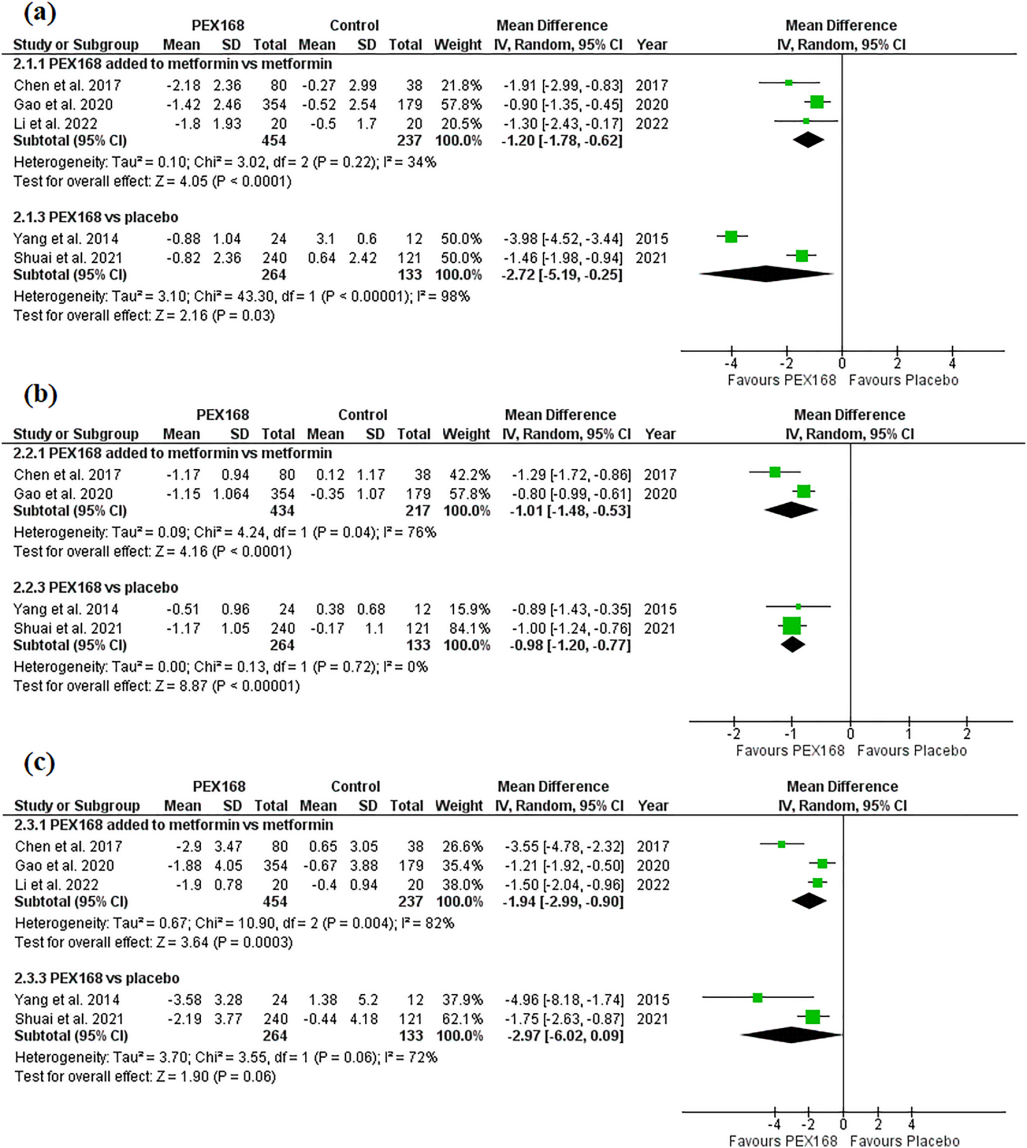
The glycemic control effects of PEX168 added to metformin vs. metformin and PEX168 monotherapy vs. placebo. Forest plots of random-effects meta-analysis are shown for (**a**) the mean difference in fasting blood glucose, (**b**) the mean difference in HbA1c, and (**c**) the mean difference in postprandial plasma glucose.

PEX168 as an add-on therapy to metformin was significantly superior to metformin in lowering FBG (MD = −1.20, 95% CI (−1.78,  − 0.62), *p* < 0.0001), HbA1c (MD = −1.01, 95% CI (−1.48,  − 0.53), *p* < 0.0001), and PPG (MD = −1.94, 95% CI (−2.99,  − 0.90), *p* = 0.0003). Pooled studies were heterogeneous for HbA1c (I^2^ = 76%, *P* = 0.04) and PPG (I^2^ = 82%, *P* = 0.004) and homogeneous for FBG (I^2^ = 34%, *P* = 0.22). PEX168 monotherapy was significantly superior to placebo in terms of FBG (MD = −2.72, 95% CI (−5.19,  − 0.25), *p* = 0.03) and HbA1c (MD = −0.98, 95% CI (−1.20,  − 0.77), *p* < 0.00001), but was statistically insignificant regarding PPG (MD = −2.97, 95% CI (−6.02, 0.09), *p* = 0.06). Pooled studies were homogeneous for HbA1c (I^2^ = 0%, *P* = 0.72) and heterogeneous for FBG (I^2^ = 98%, *P* < 0.00001) and PPG (I^2^ = 72%, *P* = 0.06). Table 3 shows the sample size and pairwise meta-analysis results for each outcome. Figure 2 shows the forest plots of the meta-analysis of FBG (a), HbA1c (b), and PPG (c) for each group.

**Table 3:** shows sample size and pairwise meta-analysis results for each outcome.

Name of the outcome	Number of the studies	Mean difference	Risk ratio	95% CI Start	95% CI End	P(Z)-value	I-Sqr (Q)	P(Q) Hetero
	(N. PEX168/ N. control)							
PEX168 + metformin versus metformin								
** HbA1c**	**2**	− **1.01**	**NA**	− **1.48**	− **0.53**	** < 0.0001**	**76**	**0.04**
	(**434/217**)							
** FBG**	**3**	− **1.2**	**NA**	− **1.78**	− **0.62**	** < 0.0001**	**34**	**0.22**
	(**454/237**)							
** PPG**	**3**	− **1.94**	**NA**	− **2.99**	− **0.9**	**0.0003**	**82**	**0.004**
	(**454/237**)							
TG	3	− 0.03	NA	− 0.33	0.27	0.86	32	0.23
	(454/237)							
LDL	3	− 0.07	NA	− 0.55	0.4	0.76	86	0.0008
	(454/237)							
HDL	3	0.04	NA	− 0.06	0.14	0.43	67	0.05
	(454/237)							
Body weight change	2	− 3.47	NA	− 10.86	3.92	0.36	90	0.002
	(374/199)							
Any AEs	2	NA	1.93	0.53	7.02	0.32	89	0.003
	(441/220)							
** Nausea**	**2**	**NA**	**4.82**	**1.14**	**20.35**	**0.03**	**0**	**0.81**
	(**441/220**)							
Vomiting	2	NA	5.9	0.78	44.9	0.09	0	0.92
	(441/220)							
Diarrhea	2	NA	3.67	0.84	16.06	0.08	0	0.4
	(441/220)							
Discontinuation of the study due to AEs	2	NA	1.02	0.3	3.44	0.97	0	0.33
	(441/220)							
PEX168 monotherapy versus placebo								
** HbA1c**	**2**	− **0.98**	**NA**	− **1.2**	− **0.77**	** < 0.0001**	**0**	**0.72**
	(**264/133**)							
** FBG**	**2**	− **2.72**	**NA**	− **5.19**	− **0.25**	**0.03**	**98**	** < 0.00001**
	(**264/133**)							
PPG	**2**	− 2.97	NA	− 6.02	0.09	0.06	72	0.06
	(**264/133**)							
	(240/121)							
Any AEs	2	NA	1.32	0.51	3.39	0.57	60	0.12
	(274/133)							
** Nausea**	**2**	**NA**	**9.88**	**1.35**	**72.01**	**0.02**	**0**	**0.41**
	(**274/135**)							
** Vomiting**	**2**	**NA**	**9.51**	**1.31**	**68.93**	**0.03**	**0**	**0.73**
	(**274/135**)							
Diarrhea	2	NA	1.67	0.25	11.25	0.6	48	0.17
	(274/135)							

Significant values are in bold.

*AEs* Adverse events; *CI* Confidence interval; *FPG* Fasting plasma glucose; *HbA1c* Glycated hemoglobin; *HDL* High-density lipoprotein; *LDL* Low-density lipoprotein; *NA* Not applicable, *PPG* Postprandial glucose; *TG* Triglyceride.

#### Lipid profiles

**Figure 3:**
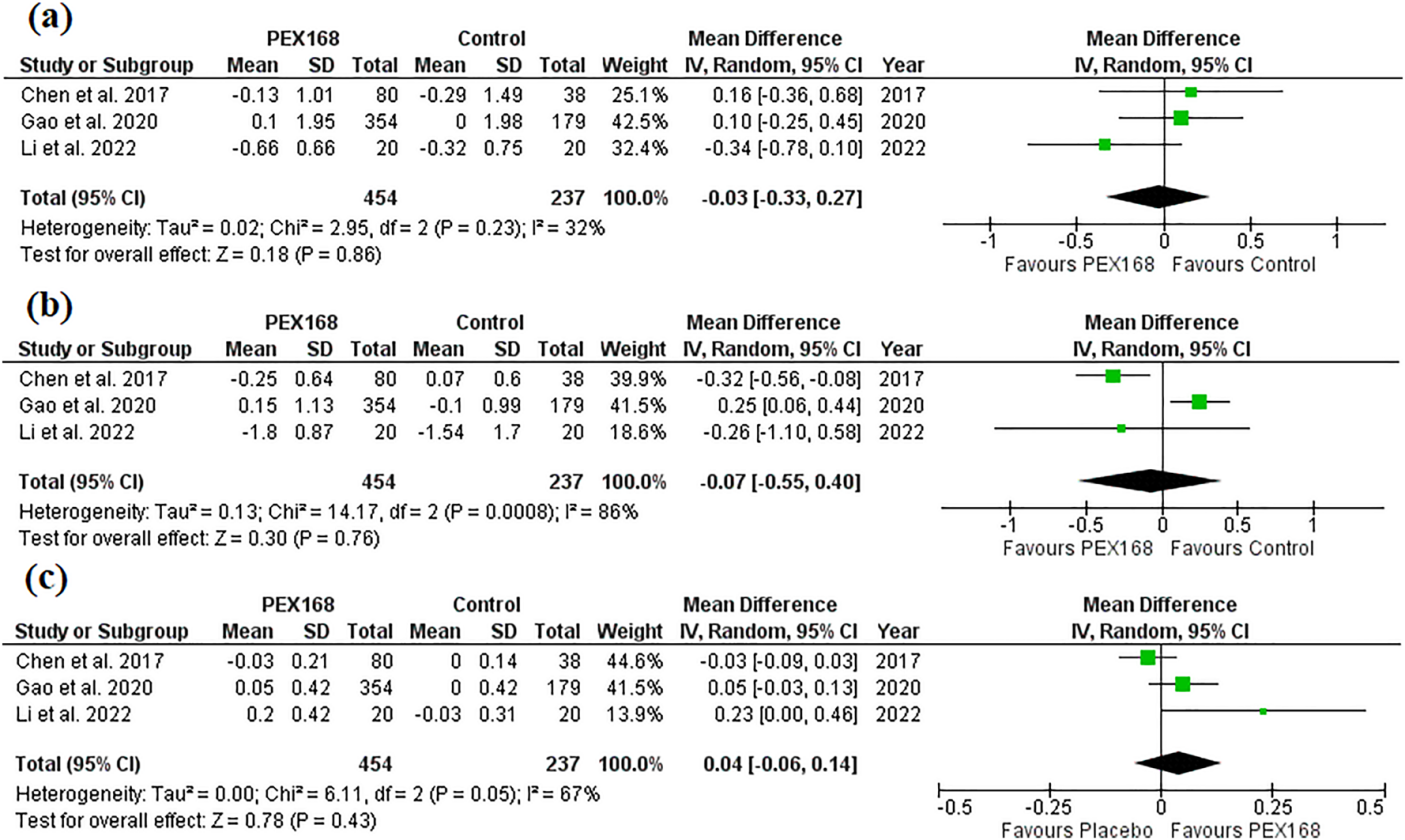
The blood lipid level effects of PEX168 added to metformin vs. metformin. Forest plots of random-effects meta-analysis are shown for (**a**) the mean difference in triglycerides, (**b**) the mean difference in low-density lipoprotein, and (**c**) the mean difference in high-density lipoprotein.

PEX168 added to metformin was similar to metformin for TG (MD = −0.03, 95% CI (−0.33, 0.27), *p* = 0.86), LDL (MD = −0.07, 95% CI (−0.55, 0.40), *p* = 0.76), and HDL (MD = 0.04, 95% CI (−0.06, 0.14), *p* = 0.43). Pooled studies were homogenous for TG (I^2^ = 32%, *P* = 0.23) and heterogenous for HDL (I^2^ = 67%, *P* = 0.05) and LDL (I^2^ = 86%, *P* = 0.0008). Table 3 shows the sample size and pairwise meta-analysis results for each outcome. Figure 3 shows the forest plots of the meta-analysis of TG (a), LDL (b), and HDL (c).

#### Body weight

**Figure 4:**
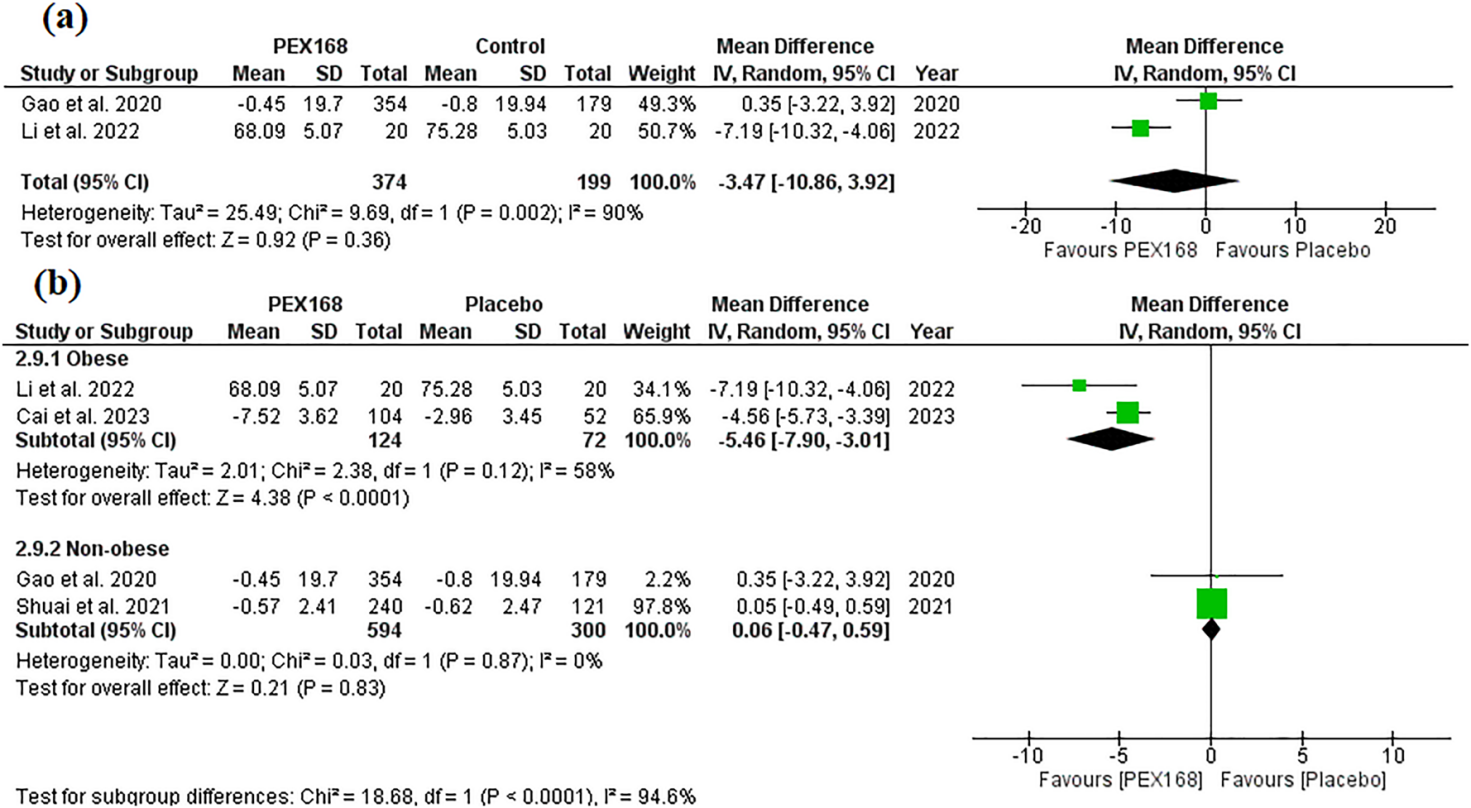
A forest plot of random-effects meta-analysis for the weight loss effects of PEX168 added to metformin vs. metformin, as shown in (**a**). A forest plot of random-effects meta-analysis for subgroup analysis of the effect of PEX168 on weight loss in obese and non-obese diabetic patients is shown in (**b**).

PEX168 as an add-on therapy to metformin was not significantly different from metformin in lowering body weight (MD = −3.47, 95% CI (−10.86, 3.92), *p* = 0.36). Pooled studies were heterogeneous (I^2^ = 90%, *P* = 0.002). Table 3 shows the sample size and pairwise meta-analysis results for each outcome. Figure 4a shows the forest plot of the meta-analysis of weight loss. However, subgrouping studies based on baseline body weight revealed that PEX168 was effective in obese patients (MD = −5.46, 95% CI (−7.90,  − 3.01), *p* < 0.0001) but insignificant in non-obese patients (MD = 0.06, 95% CI (−0.47, 0.59), *p* = 0.83). Pooled studies were homogenous for both subgroups (I^2^ = 58%, *P* = 0.12) and (I^2^ = 0%, *P* = 0.87). Figure 4b shows the forest plot of the meta-analysis of weight loss for obese and non-obese diabetic patients.

#### Safety

**Figure 5:**
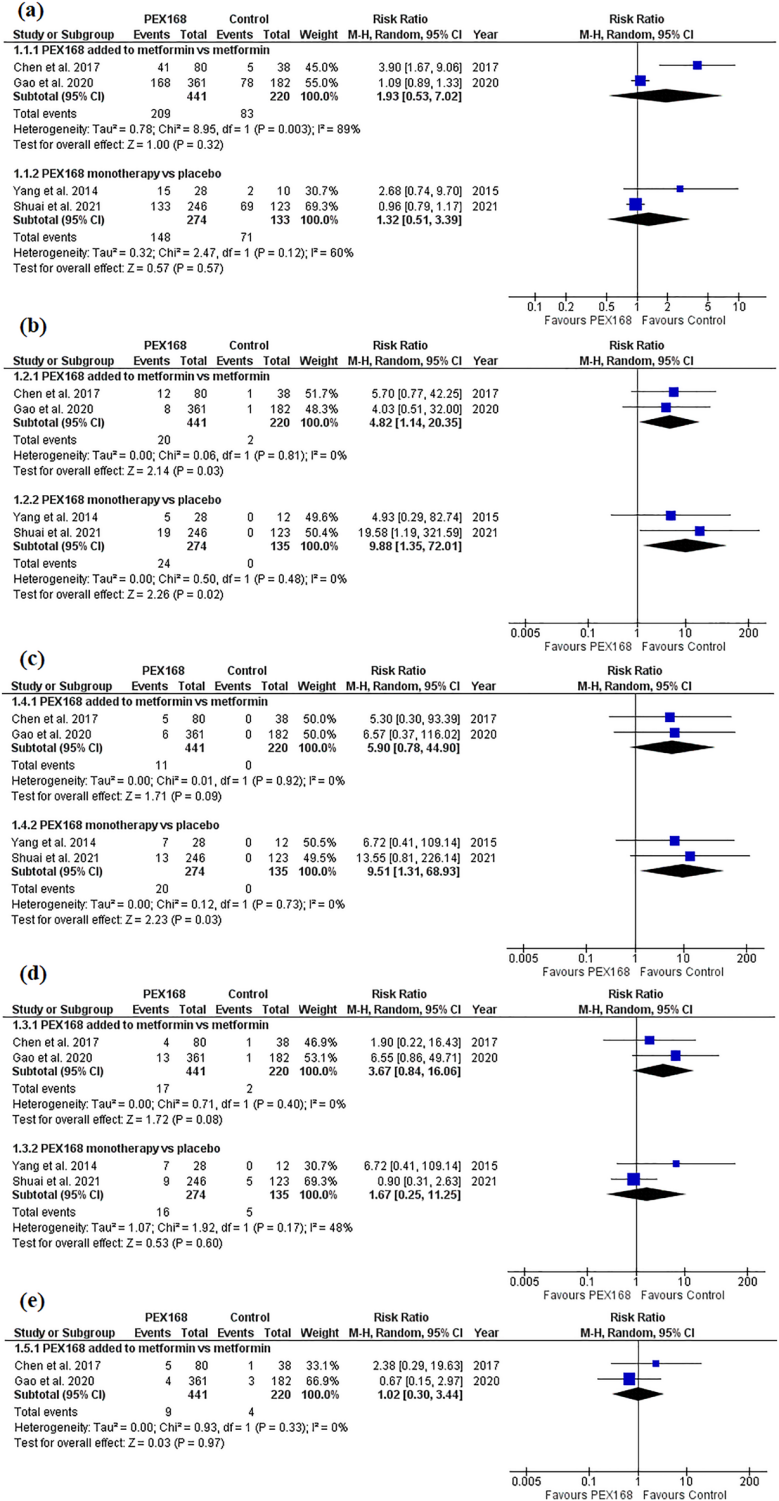
The incidences of the adverse events of PEX168 added to metformin vs. metformin and PEX168 monotherapy vs. placebo. Forest plots of random-effects meta-analysis are shown for (**a**) the risk ratio of any adverse events (AEs), (**b**) the risk ratio of nausea, (**c**) the risk ratio of vomiting, (**d**) the risk ratio of diarrhea, and (**e**) the risk ratio of discontinuation of the study due to AEs.

In the pairwise meta-analysis, PEX168 added to metformin showed a significant incidence of nausea compared to metformin alone (RR = 4.82, 95% CI (1.14, 20.35), *p* = 0.03), while the incidence of vomiting (RR = 5.90, 95% CI (0.78, 44.90), *p* = 0.09), diarrhea (RR = 3.67, 95% CI (0.84, 16.06), *p* = 0.08), any adverse events (AEs) (RR = 1.93, 95% CI (0.53, 7.02), *p* = 0.32), and discontinuation of the study due to AEs (RR = 1.02, 95% CI (0.30, 3.44), *p* = 0.97) were insignificant. Pooled studies were homogeneous for all outcomes (I^2^ = 0%, *P* > 0.10) except any AEs (I^2^ = 89%, *P* = 0.003). Compared to placebo, PEX168 monotherapy significantly raised the risk of nausea and vomiting (RR = 9.88, 95% CI (1.35, 72.01), *p* = 0.02), (RR = 9.51, 95% CI (1.31, 68.93), *p* = 0.03), while the incidences of any AEs (RR = 1.32, 95% CI (0.51, 3.39), *p* = 0.57), and diarrhea (RR = 1.67, 95% CI (0.25, 11.25), *p* = 0.60). Pooled studies were homogeneous for all outcomes (I^2^ < 60%, *P* > 0.10). Table 3 shows the sample size and pairwise meta-analysis results for each outcome. Figure 5 shows the forest plots of the meta-analysis of AEs (a), nausea (b), vomiting (c), diarrhea (d), and (e) discontinuation of the study due to AEs for each group.

#### Network meta-analysis findings

**Figure 6:**
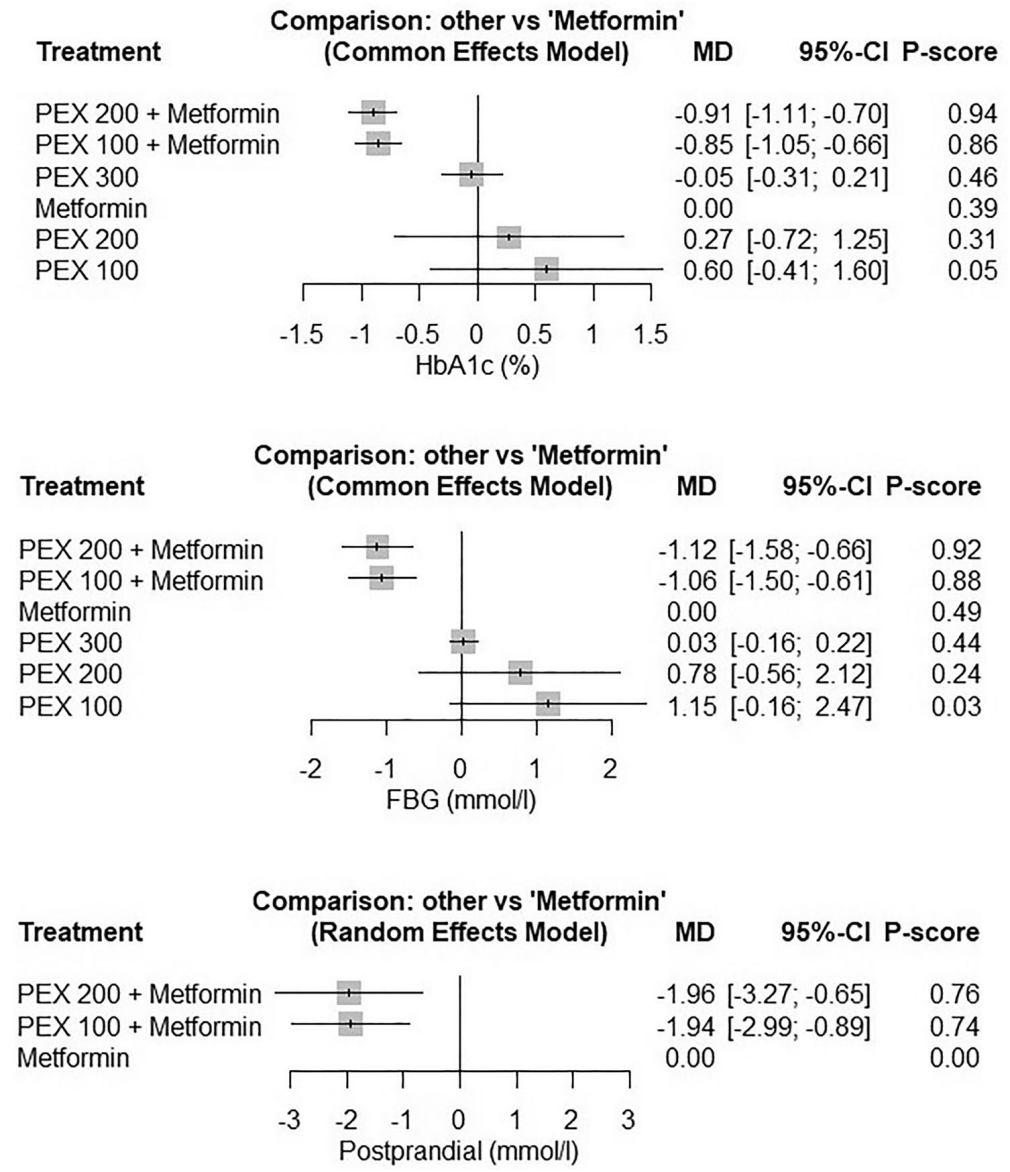
The glycemic control effects of the different PEX168 doses compared to metformin. Forest plots of network meta-analysis are shown for the pooled mean difference of the different PEX168 doses compared to metformin for HbA1c, fasting blood glucose (FBG), and postprandial plasma glucose (PPG).

The NMA showed that the PEX 200 and 100 added to metformin significantly decreased the HbA1c (MD = −0.91, 95% CI [−1.11,  − 0.70]), (MD = −0.85, 95% CI [−1.05,  − 0.66]), FBG (MD = −1.12, 95% CI [−1.58,  − 0.66]), (MD = −1.06, 95% CI [−1.50,  − 0.61]), and PPG (MD = −1.96, 95% CI [−3.27,  − 0.65]), (MD = −1.94, 95% CI [−2.99,  − 0.89]). PEX 300, PEX 200, and PEX 100 monotherapy did not significantly differ from metformin regarding HbA1c, FBG, and PPG. The heterogeneity was insignificant for HbA1c (I^2^ = 50%, *P* = 0.10) and FBG (I^2^ = 0%, *P* = 0.53) and significant for PPG (I^2^ = 74%, *P* = 0.008). Figure 6 shows the forest plots and the ranking of the PEX168 doses from highest to lowest efficacy according to the p-score. Figures S2–S7 show network plots and head-to-head comparisons among the included interventions for HbA1c, FBG, and PPG.

**Figure 7:**
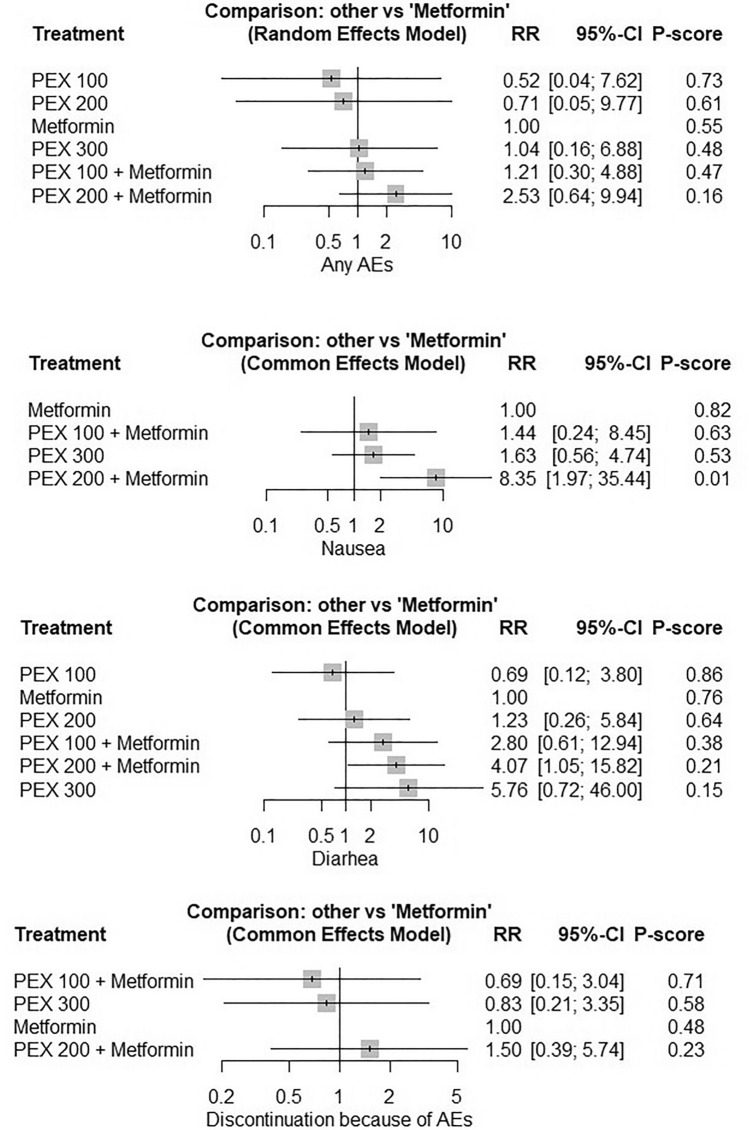
The incidences of adverse events of the different PEX168 doses compared to metformin. Forest plots of network meta-analysis are shown for the pooled risk ratios of the different PEX168 doses compared to metformin for any adverse events (AEs), nausea, diarrhea, and discontinuation of the study due to AEs.

The NMA showed that PEX 300 ug monotherapy and PEX 100 and PEX 200 combined with metformin resulted in higher rates of AEs, nausea, and diarrhea. However, only PEX 200 added to metformin approached statistical significance regarding nausea (RR = 8.35, 95% CI [1.97, 35.44]) and diarrhea (RR = 4.07, 95% CI [1.05, 15.82]). The heterogeneity was significant for any AEs (I^2^ = 90%, *P* < 0.0001) and insignificant for diarrhea (I^2^ = 0%, *P* = 0.44), nausea (I^2^ = 0%, *P* = 0.94), and discontinuation of the study due to AEs (I^2^ = 0%, *P* = 0.40). PEX 100 ug caused fewer AEs and diarrhea, but there was no statistical significance (*P* > 0.05). Figure 7 shows the forest plots and the ranking of the included interventions from highest to lowest safety according to the p-score. Figures S8–S15 show network plots and head-to-head comparisons. We could not conduct NMA on the incidence of vomiting due to the absence of events across several arms.

#### Assessment of publication bias

The funnel plot did not rule out publication bias, as shown in (Figure S16). Egger's (*P* = 0.077) and Begg's (*P* = 0.57) tests revealed no evidence of publication bias among the included studies.

## Discussion

In this systematic review and meta-analysis, we assessed the efficacy and safety of PEX168 as monotherapy or add-on therapy to metformin in type 2 diabetic patients. Our findings suggest that PEX168, both as monotherapy and as an add-on to metformin, demonstrated significant improvements in glycemic control compared to placebo and metformin alone. Furthermore, PEX168 showed a beneficial effect on body weight reduction in patients with obesity. However, some safety concerns were identified, particularly gastrointestinal adverse effects.

Glycemic control outcomes revealed that PEX168 as an add-on therapy to metformin significantly reduced HbA1c, FBG, and PPG levels compared to metformin alone. The findings were consistent for the three clinical trials that made this comparison^25, 27, 28^. PEX168 monotherapy was superior to placebo in terms of HbA1c and FBG. These findings indicate that PEX168 might be a viable treatment option for patients with inadequate glycemic control on metformin or those who cannot tolerate metformin. The network meta-analysis results further support these findings, with PEX168 200 ug plus metformin and PEX168 100 ug plus metformin showing the most substantial reduction in HbA1c, FBG, and PPG levels.

Elevated TG and LDL levels have been linked to the progression of atherosclerosis and other cardiovascular complications, emphasizing the significance of addressing lipid abnormalities in diabetes management^35, 36^. According to our meta-analysis, adding PEX168 to metformin had no significant effect compared to metformin alone. This finding was consistent across the three clinical trials included in this comparison. Reducing TG and LDL levels offers potential benefits for mitigating the cardiovascular burden of individuals with type 2 diabetes. Lowering TG levels could lead to decreased atherogenic risk^36^, while the decrease in LDL levels may contribute to attenuating atherosclerotic plaque formation^35, 37^. Cai et al.^26^ compared PEX168 monotherapy to metformin, revealing that PEX168 monotherapy significantly reduced TG and LDL. Cai et al.^26^ had the lowest baseline triglyceride levels, the highest baseline BMI, and remarkable weight loss at the end of the study, all of which correlated with the high efficacy of the similarly structured exenatide on cardiovascular risk factors^38, 39^. Therefore, the effect of PEX168 on lipid profiles may be related to patient characteristics rather than the treatment regimen. However, further investigation is needed to confirm this and better understand the implications of PEX168 on lipid profiles and cardiovascular outcomes in type 2 diabetic patients, which will help guide clinical decisions.

Regarding body weight reduction, PEX168 was superior to metformin in reducing body weight in obese patients but not in non-obese patients. This finding is in line with earlier research, where it was found that patients with the highest body weight benefited most from the weight loss effects of GLP-1RAs^39^. This finding could be related to differences in underlying pathophysiology or metabolic adaptations in non-obese individuals. Additionally, factors such as adherence to treatment, dietary habits, and physical activity levels could contribute to varying treatment responses. Further research is needed to elucidate the specific reasons behind this observation and identify potential treatment response predictors in terms of body weight reduction. These findings highlight the potential benefits of PEX168 monotherapy in managing body weight in type 2 diabetic patients with obesity, which may offer additional benefits in managing obesity-related complications in type 2 diabetic patients. Furthermore, obesity is a significant risk factor for developing type 2 diabetes associated with increased insulin resistance^40^. The potential for more substantial weight loss in the obese subgroup could translate to improved glycemic control and reduced insulin resistance, which are critical factors in diabetes management. Our findings also imply that non-obese patients can benefit from the glycemic control of PEX168 without the risk of excessive weight loss. A similar finding was reported for the similarly structured exenatide^41^.

Because statistical and clinical significance should be interpreted differently, it is critical to emphasize that PEX168 had a clinical and statistically significant effect. According to the literature, the minimum clinically significant difference for FBG and HbA1c is 0.5 mmol/L^42^ and 0.5%^43, 44^, respectively, and the minimum clinically significant difference for body weight loss is 5% of body weight^45^. In the pairwise meta-analysis, PEX168 as an add-on therapy reduced FPG and HbA1c by 1.2 mmol/L and 1.01% compared to metformin, and it reduced FBG and HbA1c as monotherapy by 2.72 mmol/L and 0.98% compared to placebo. This was confirmed by a network meta-analysis, which found that both doses of PEX168 as add-on therapy, 200 ug and 100 ug, produced clinically significant differences when compared to metformin alone in terms of FBG:  − 0.91,  − 0.85, and HbA1c:  − 1.12,  − 1.06, respectively. Furthermore, PEX168 reduced body weight in obese patients by 5.46 kg, greater than 5% of the patients' baseline body weight. This finding is consistent with the findings of Cai et al. 2023 who found that the proportion of patients who received PEX168 and lost more than 5% and 10% of their body weight was significantly higher than those who received metformin. Therefore, PEX168 effects on the reduction of glycemic parameters and body weight were clinically meaningful and statistically significant.

Our study also assessed the safety of PEX168 and found that, apart from PEX 100 ug monotherapy, which was relatively safer than metformin, PEX168 increased the risk of gastrointestinal adverse effects, particularly nausea and vomiting, when used as an add-on therapy to metformin or as a monotherapy compared to the control group. However, the overall incidence of adverse events and discontinuation of the study due to adverse events were not significantly different between the groups. Clinicians should carefully consider these potential adverse effects when prescribing PEX168 and monitor patients for any adverse reactions. PEX168 increased the occurrence of adverse events in a dose-dependent manner, with PEX168 200 ug combined with metformin performing the worst and having a significantly higher prevalence of nausea and diarrhea compared to metformin. Therefore, the combination of PEX168 100 ug and metformin may be the optimal regimen for safety and efficacy; however, results should be interpreted with caution due to the small number of RCTs included.

Current guidelines for type II diabetes management emphasize a patient-centered approach that includes lifestyle modifications, pharmacologic therapy, and regular monitoring to achieve individualized glycemic targets^46, 47^. Metformin is typically the first-line medication for most patients due to its proven efficacy, safety profile, and cost-effectiveness. Suppose metformin alone does not achieve adequate glycemic control or is not tolerated. In that case, additional medications such as sulfonylureas, DPP-4 inhibitors, SGLT2 inhibitors, or GLP-1 receptor agonists may be considered based on the patient's needs and risk factors^46, 47^.

This meta-analysis has shed light on the potential benefits of PEX168 as monotherapy or as an add-on therapy to metformin for type II diabetes patients, showing significant improvements in glycemic control and body weight reduction. These findings may prompt future revisions of clinical guidelines to consider incorporating PEX168 as a viable treatment option for patients with inadequate glycemic control on metformin or those who cannot tolerate metformin. Furthermore, PEX168 may play a special role in obese patients by regulating body weight and potentially reducing related complications. However, the gastrointestinal adverse effects of PEX168 should be considered when making treatment decisions. As more research emerges to support the long-term safety and efficacy of PEX168, it is possible that this medication could become a valuable addition to the current treatment options for type II diabetes management, ultimately leading to improved patient outcomes and quality of life.

## Limitations

There are some limitations. The quality of the included studies varied, with one study having a high risk of bias^24^, which might affect the reliability of the findings. Additionally, the available data were limited by the small number of studies and short and different follow-up periods, which may not fully capture long-term treatment effects or potential safety concerns.

Future studies should focus on evaluating the long-term safety and efficacy of PEX168 in type 2 diabetes management, exploring potential dose-dependent effects, and comparing PEX168 with other emerging therapies. Larger sample sizes and longer follow-up durations should be considered in future studies to address the limitations of the current evidence. Additionally, research should investigate the relationship between GLP-1RAs and patient lipid profiles, which could help optimize treatment strategies and identify potential biomarkers for treatment response. Moreover, studies should also evaluate the cost-effectiveness of PEX168 compared to other treatment options and its impact on patient-reported outcomes and quality of life.

## Conclusions

Our systematic review and meta-analysis suggest that PEX168 as monotherapy or add-on therapy to metformin may be a promising treatment option for glycemic control in type 2 diabetic patients with inadequate control on metformin or those who cannot tolerate metformin, as well as body weight management in type 2 diabetic patients with obesity; however, due to the small number of RCTs included, this should be interpreted with caution and further trials are needed. More research is required to fully understand the implications of PEX168 on lipid profiles and identify the variables correlated with patient responses. Furthermore, the potential gastrointestinal adverse effects warrant careful consideration. Current evidence suggests that PEX168 at 100 ug in combination with metformin is the optimal dose; however, further research is needed to confirm or refute these findings and assess the long-term safety and efficacy of PEX168 in type 2 diabetes management.

### Supplementary Information

Supplementary Information.

## Data Availability

All data analyzed during this study are included in this published article or listed in references.

## Abbreviations

AEs: Adverse events
BMI: Body mass index
CI: Confidence intervals
FPG: Fasting plasma glucose
GLP-1Ras: Glucagon-like peptide 1 receptor agonists
HbA1c: Glycated hemoglobin
HDL: High-density lipoprotein
LDL: Low-density lipoprotein
MD: Mean difference
NMA: Network meta-analysis
PEG: Polyethylene glycol
PEX168: Polyethylene glycol loxenatide
PRISMA: Preferred Reporting Items for systematic reviews and meta-analyses
RCTs: Randomized controlled trials
ROB: Risk of bias
RR: Risk ratio
SD: Standard deviation
T2DM: Type 2 diabetes mellitus
TG: Triglyceride
Ug: Microgram
