# Supplementary material for: Efficacy and safety of polyethylene glycol loxenatide in type 2 diabetic patients: a systematic review and meta-analysis of randomized controlled trials
Source: Sci Rep. 2023 Nov 3;13:19041. doi: 10.1038/s41598-023-46274-x (PMC10624877; doi:10.1038/s41598-023-46274-x)
Supplement: Supplementary file 1 — Supplementary Information. [file 41598_2023_46274_MOESM1_ESM.docx]

**Contents:**

Table S1: Full search strategy for each database.

Figure S1: Summary of the assessment of the risk of bias.

Figure S2: Network plot of HbA1c.

Figure S3: Forest plot of head-to-head comparisons of HbA1c.

Figure S4: Network plot of FBG.

Figure S5: Forest plot of head-to-head comparisons of FBG.

Figure S6: Network plot of PPG.

Figure S7: Forest plot of head-to-head comparisons of PPG.

Figure S8: Network plot of AEs.

Figure S9: Forest plot of head-to-head comparisons of AEs.

Figure S10: Network plot of nausea.

Figure S11: Forest plot of head-to-head comparisons of nausea.

Figure S12: Network plot of diarrhea.

Figure S13: Forest plot of head-to-head comparisons of diarrhea.

Figure S14: Network plot of discontinuation of the study due to AEs.

Figure S15: Forest plot of head-to-head comparisons of discontinuation of the study due to AEs.

Figure S16: The funnel plot of publication bias of the included studies.

| Database | Search Terms | Search Field | Search Results |
| --- | --- | --- | --- |
| PubMed | "PEX168" OR "PEX 168" OR "PEX-168" OR "PEG-Loxe" OR "PEG-loxenatide" OR "polyethylene glycol loxenatide" OR "loxenatide" | All Fields | 16 |
| Cochrane | "PEX168" OR "PEX 168" OR "PEX-168" OR "PEG-Loxe" OR "PEG-loxenatide" OR "polyethylene glycol loxenatide" OR "loxenatide" | All Fields | 17 |
| WOS | "PEX168" OR "PEX 168" OR "PEX-168" OR "PEG-Loxe" OR "PEG-loxenatide" OR "polyethylene glycol loxenatide" OR "loxenatide" | All Fields | 18 |
| SCOPUS | "PEX168" OR "PEX 168" OR "PEX-168" OR "PEG-Loxe" OR "PEG-loxenatide" OR "polyethylene glycol loxenatide" OR "loxenatide" | Title, Abstract, Keywords | 20 |

Table S1: Full search strategy for each database.


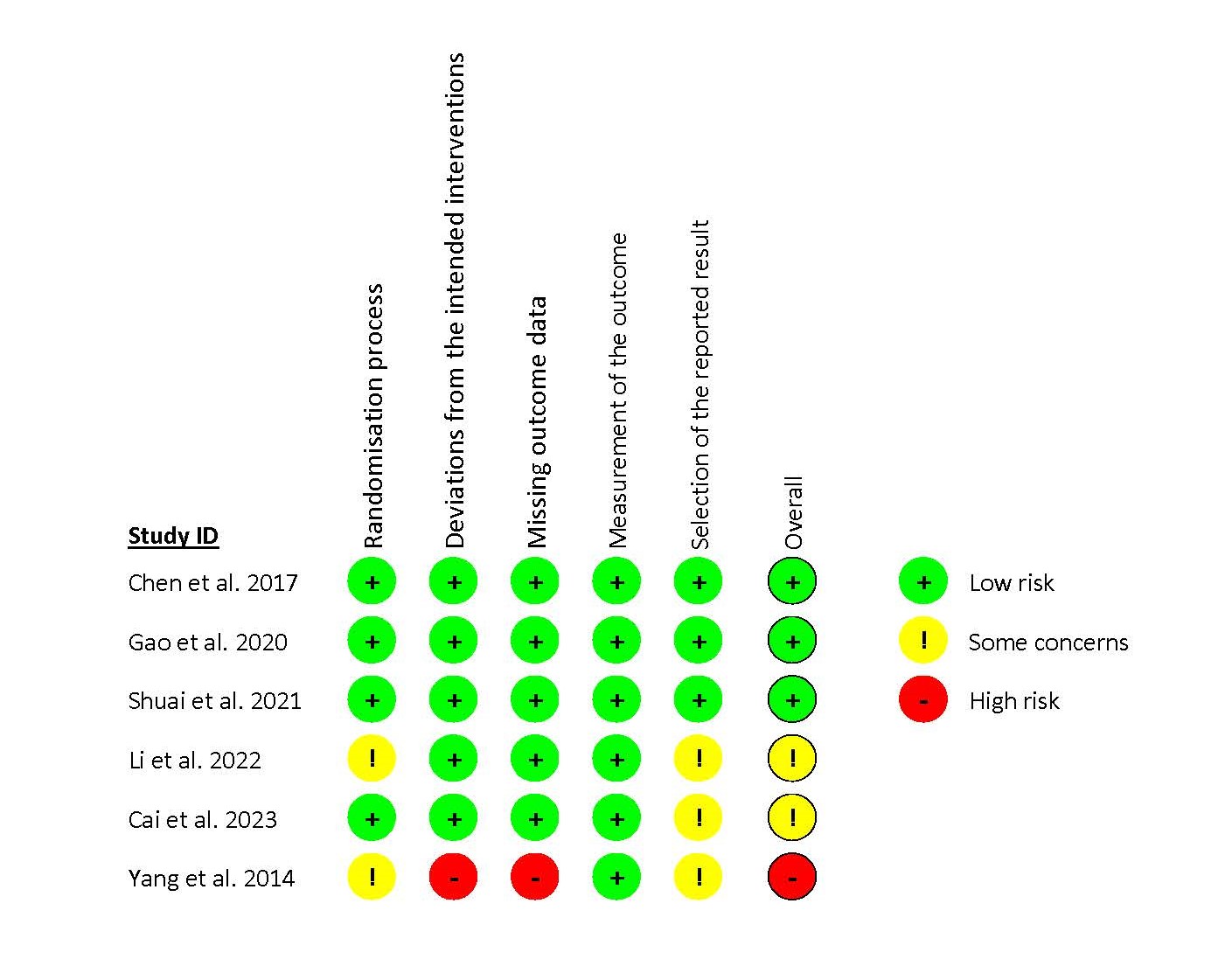


Figure S1: Summary of the assessment of the risk of bias.


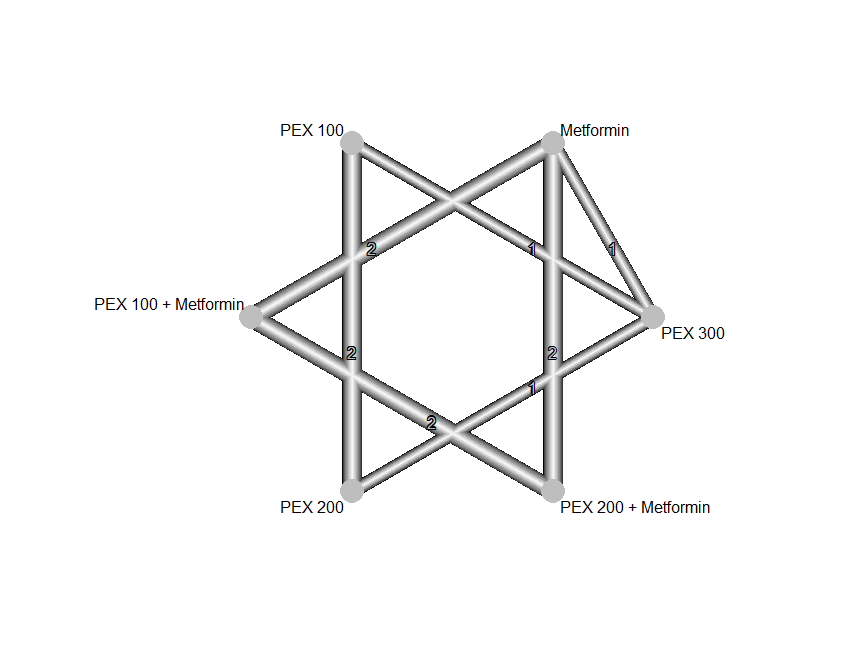


Figure S2: Network plot of HbA1c.


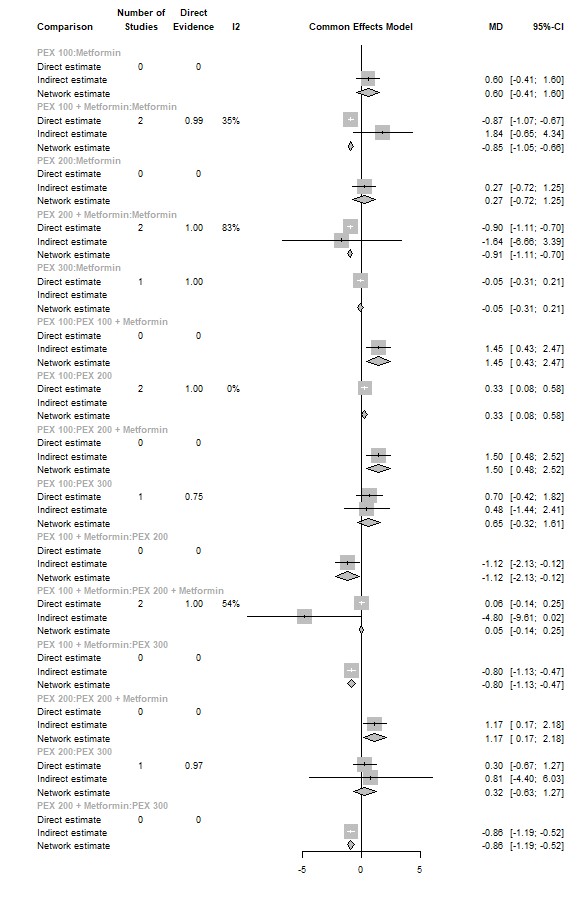


Figure S3: Forest plot of head-to-head comparisons of HbA1c


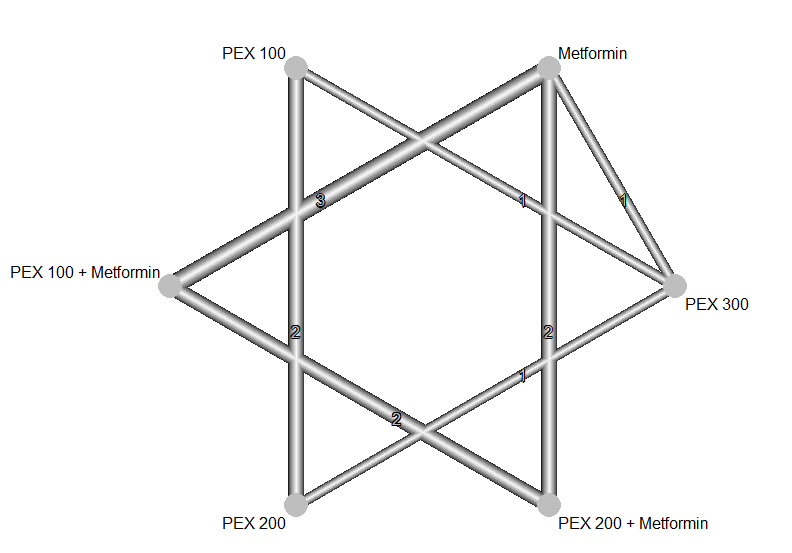


Figure S4: Network plot of FBG.


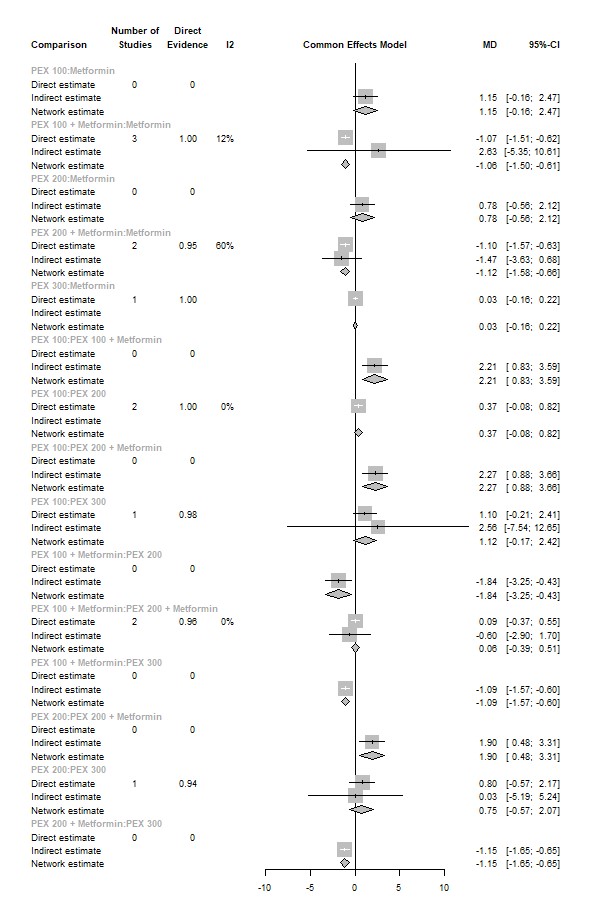


Figure S5: Forest plot of head-to-head comparisons of FBG


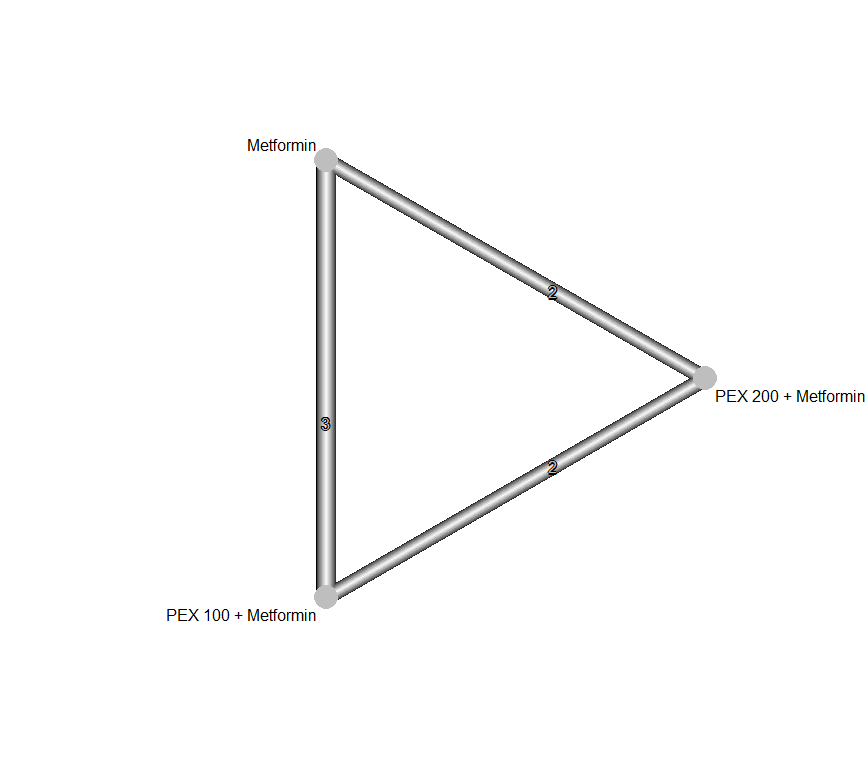


Figure S6: Network plot of PPG.


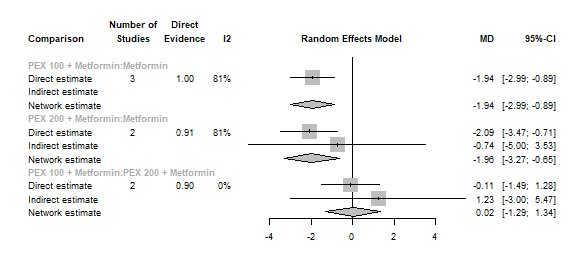


Figure S7: Forest plot of head-to-head comparisons of PPG.


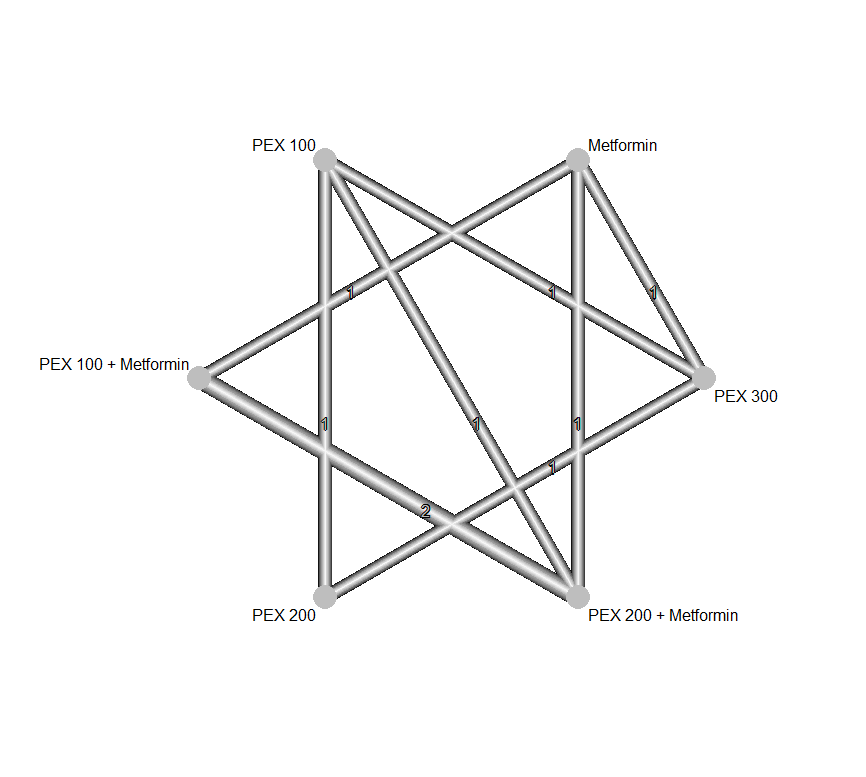


Figure S8: Network plot of AEs.


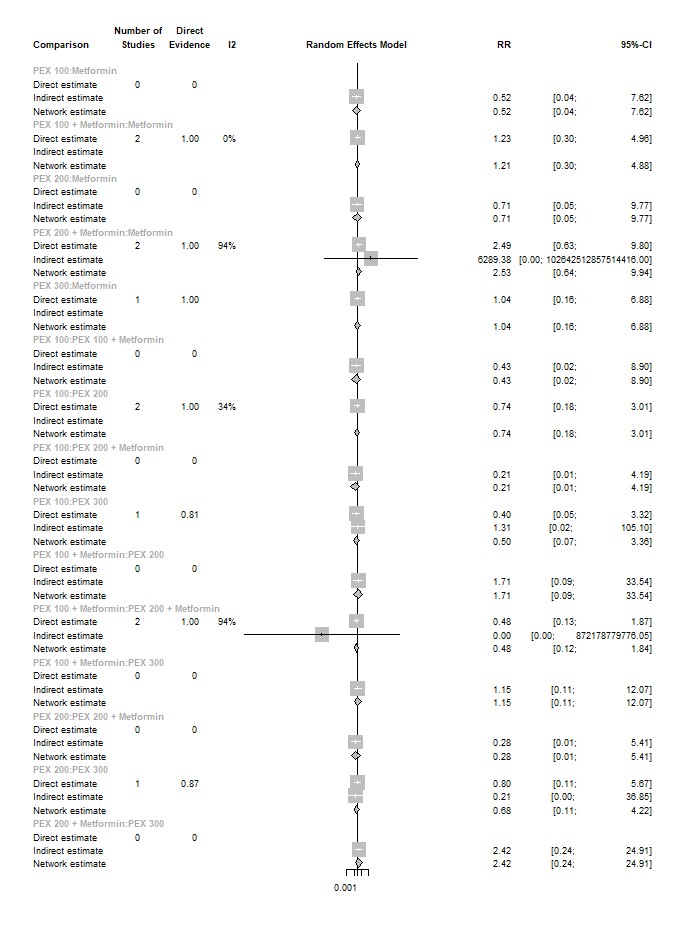


Figure S9: Forest plot of head-to-head comparisons of AEs


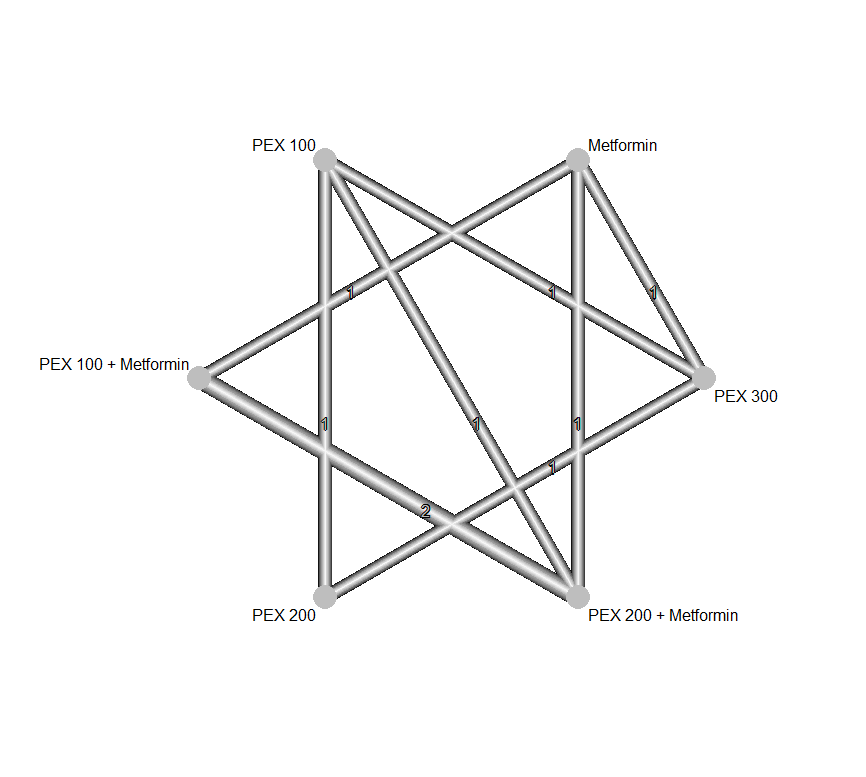


Figure S10: Network plot of nausea.


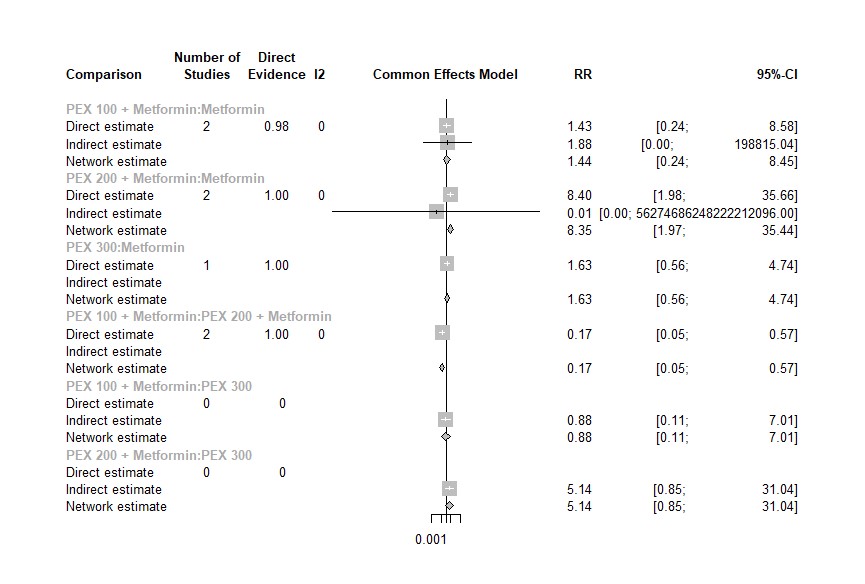


Figure S11: Forest plot of head-to-head comparisons of nausea.


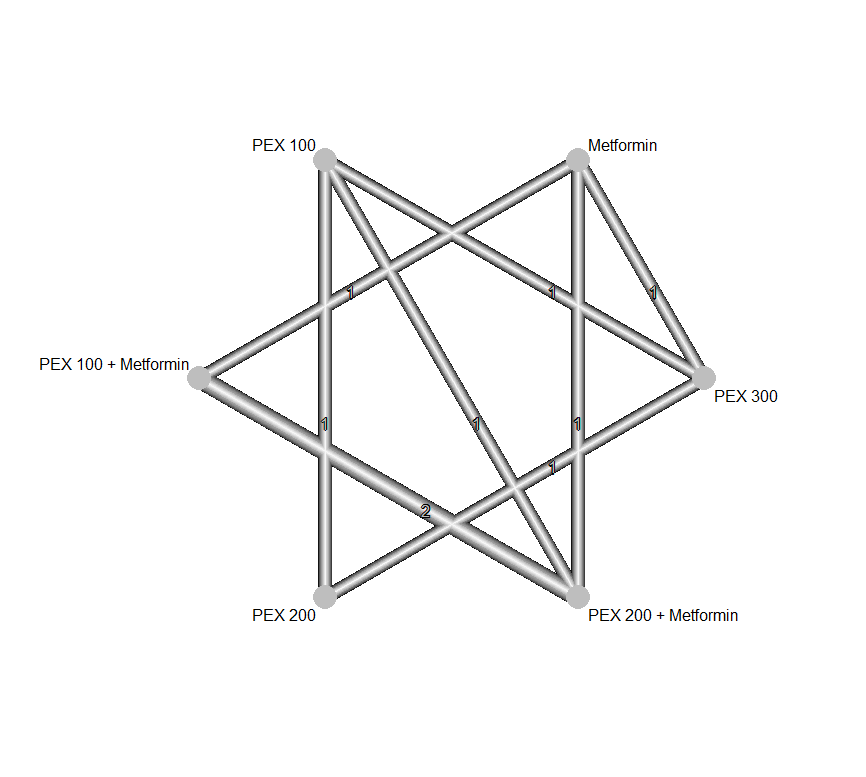


Figure S12: Network plot of diarrhea.


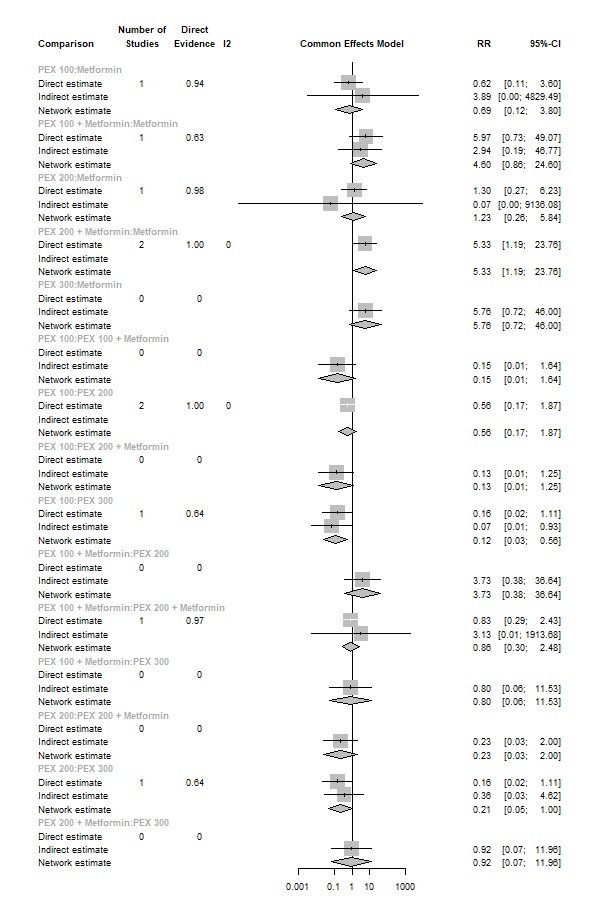


Figure S13: Forest plot of head-to-head comparisons of diarrhea.


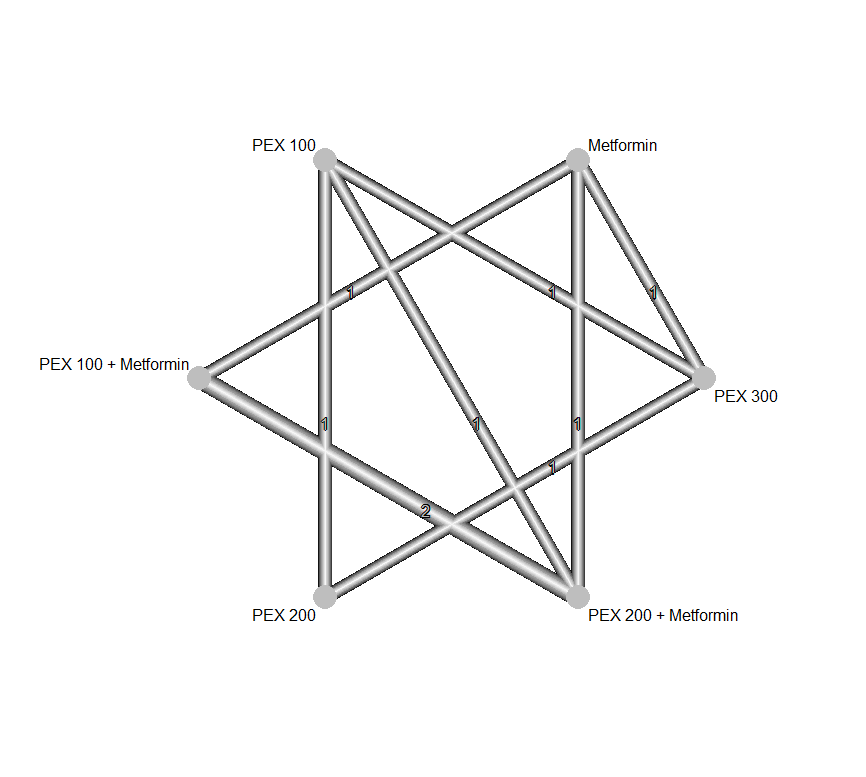


Figure S14: Network plot of discontinuation of the study due to AEs.


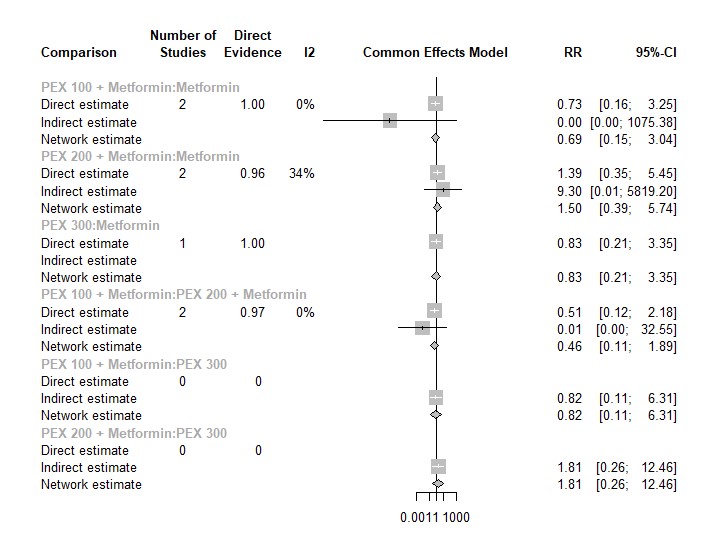


Figure S15: Forest plot of head-to-head comparisons of discontinuation of the study due to AEs.


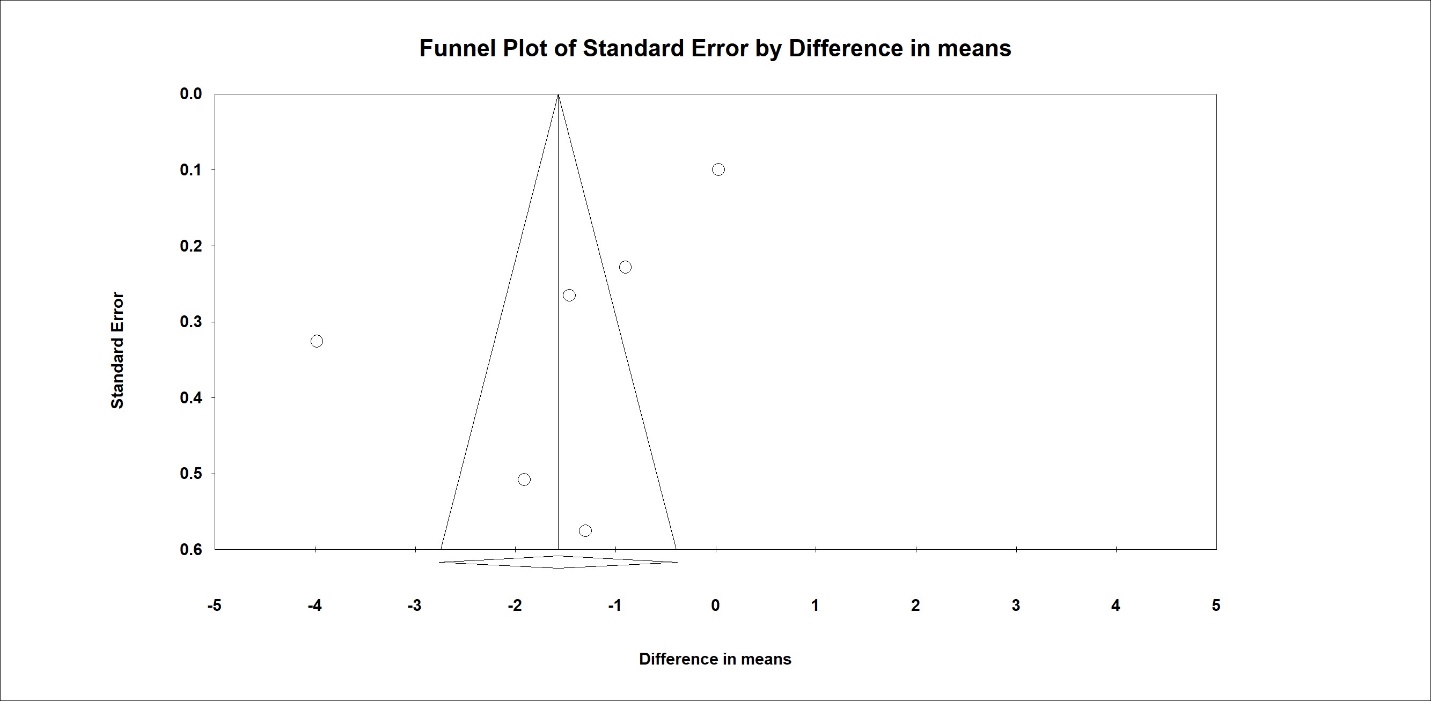


Figure S16: The funnel plot of publication bias of the included studies
